# Spc1 regulates the signal peptidase-mediated processing of membrane proteins

**DOI:** 10.1242/jcs.258936

**Published:** 2021-07-09

**Authors:** Chewon Yim, Yeonji Chung, Jeesoo Kim, IngMarie Nilsson, Jong-Seo Kim, Hyun Kim

**Affiliations:** 1 School of Biological Sciences and Institute of Microbiology, Seoul National University, Seoul 08826, South Korea; 2 Center for RNA Research, Institute for Basic Science, Seoul 08826, South Korea; 3 Department of Biochemistry and Biophysics, Stockholm University, SE-10691 Stockholm, Sweden

**Keywords:** SPCS1, Signal peptidase, Signal sequence, Spc1, Transmembrane

## Abstract

Signal peptidase (SPase) cleaves the signal sequences (SSs) of secretory precursors. It contains an evolutionarily conserved membrane protein subunit, Spc1, that is dispensable for the catalytic activity of SPase and whose role remains unknown. In this study, we investigated the function of yeast Spc1. First, we set up an *in vivo* SPase cleavage assay using variants of the secretory protein carboxypeptidase Y (CPY) with SSs modified in the N-terminal and hydrophobic core regions. When comparing the SS cleavage efficiencies of these variants in cells with or without Spc1, we found that signal-anchored sequences became more susceptible to cleavage by SPase without Spc1. Furthermore, SPase-mediated processing of model membrane proteins was enhanced in the absence of Spc1 and was reduced upon overexpression of Spc1. Spc1 co-immunoprecipitated with proteins carrying uncleaved signal-anchored or transmembrane (TM) segments. Taken together, these results suggest that Spc1 protects TM segments from SPase action, thereby sharpening SPase substrate selection and acting as a negative regulator of the SPase-mediated processing of membrane proteins.

## Abstract (precis)

**Summary:** Spc1 protects membrane proteins from being cleaved by signal peptidase, implicating a role in substrate sorting for signal peptidase.

## INTRODUCTION

Signal peptidase (SPase) is an evolutionarily conserved protease that cleaves signal sequences (SSs) of secretory precursors targeted to the plasma membrane in prokaryotes or the endoplasmic reticulum (ER) in eukaryotes. Processing occurs co- or post-translationally when a nascent chain passes through the Sec translocon (Lyko et al., 1995; Wollenberg and Simon, 2004).

In prokaryotes, SPase I (leader peptidase) functions as a monomer, whereas eukaryotic SPase is a heterooligomer consisting of membrane protein subunits, all of which are conserved from yeast to humans (Spc1 in yeast, SPCS1 in humans; Spc2 in yeast, SPCS2 in humans; Spc3 in yeast, SPCS3 in humans; and Sec11 in yeast, SEC11A and SEC11C in humans) (Antonin et al., 2000; Dalbey et al., 2017; Dalbey and Von Heijne, 1992; Evans et al., 1986; Fang et al., 1996; Greenburg et al., 1989; Shelness and Blobel, 1990; YaDeau et al., 1991; Zwizinski and Wickner, 1980).

In yeast, Sec11 and Spc3 are required for the catalytic activity of eukaryotic SPase and are essential for cell viability. Both Sec11 and Spc3 are single-pass membrane proteins with the C-terminal domain facing the lumen, and their luminal domains exhibit sequence homology to the leader peptidase active domain (Fang et al., 1997; VanValkenburgh et al., 1999). Spc2 has been found to associate with a beta subunit of the Sec61 complex in both yeast and mammals, suggesting a role in an interaction between the SPase complex and the Sec61 translocon (Antonin et al., 2000; Kalies et al., 1998).

Spc1 was first identified in a homology search using mammalian SPCS1 (also known as SPC12) and by genetic interaction with Sec11 in yeast (Fang et al., 1996). Although Spc1 is dispensable for cell viability in yeast, deletion of SPC12 (Spc1 homolog, also known as Spase12) in *Drosophila* causes a developmental defect, indicating a crucial role in higher eukaryotes (Haase Gilbert et al., 2013). In the yeast strain lacking Spc1, signal peptides of secretory precursors are efficiently cleaved (Fang et al., 1996; Mullins et al., 1996); hence, the role of Spc1 in SPase remains puzzling*.*

SSs have distinctive characteristics: a hydrophobic core (*h* region) containing consecutive nonpolar amino acids, which can form at least two turns in an α-helix, is flanked by N-terminal (*n* region) and C-terminal (*c* region) polar and charged residues (von Heijne, 1985). Although this tripartite structure is found in all SSs, the overall and relative lengths of the *n*, *h* and *c* regions; the hydrophobicity of the *h* region; and the distribution of charged residues in the *n* and *c* regions greatly vary among them, making SSs uniquely diverse (Choo and Ranganathan, 2008; Choo et al., 2008; Gierasch, 1989; Hegde and Bernstein, 2006; von Heijne, 1985).

SPase recognizes a cleavage motif that includes small, neutral amino acids at the −3 and −1 positions relative to the cleavage site (Bird et al., 1990; Dalbey and Von Heijne, 1992; von Heijne, 1990). The structure of bacterial SPase shows binding pockets for small residues in the active site (Paetzel et al., 1998). However, not all SSs with an optimal cleavage site are processed by SPase (Nilsson et al., 1994; Yim et al., 2018). On the other hand, a signal anchor of sucrase-isomaltase has been found to be cleaved when a single amino acid is substituted within the signal anchor sequence, illustrating that subtle changes in and around the transmembrane (TM) domain can induce processing by SPase (Hegner et al., 1992). These observations imply that SPase recognizes certain characteristics in SSs in addition to the cleavage site, yet it remains unknown how SPase-mediated processing is regulated.

To investigate how SSs are sorted, and to what extent SPase mediates the process, we first set up an *in vivo* SPase cleavage assay in the yeast *Saccharomyces cerevisiae* using carboxypeptidase Y (CPY) variants carrying SSs of systematically varied length and hydrophobicity. Using this approach, we defined the substrate range of SPase in terms of *n* and *h* region features of SSs in yeast.

Next, we explored the role of Spc1. We assessed and compared the SS cleavage efficiencies of the CPY variants in cells with or without Spc1. We observed that membrane-anchored, internal SSs were more efficiently cleaved in the absence of Spc1. Mutagenesis analysis at the cleavage site showed that recognition and usage of cleavage sites by SPase was unaffected with or without Spc1. Furthermore, cleavage of a TM segment in model membrane proteins was enhanced in the absence of Spc1 but was reduced upon overexpression of Spc1.

Collectively, our data show that SPase selects substrates based on the *n* and *h* regions of the signal sequence and becomes more prone to include signal-anchored and TM domains for processing in the absence of Spc1. These results suggest that Spc1 protects TM segments of membrane proteins from being cleaved by SPase, implicating Spc1 in the regulation of substrate sorting for SPase.

## RESULTS

### Defining the substrate spectrum of SPase in *S. cerevisiae*

Previously, we observed that the secretory protein CPY with a modified hydrophobic SS [CPY(*h*); *h* for high hydrophobicity] and the same CPY precursor with an N-terminal extension [N26CPY(*h*)] localize differently (Yim et al., 2018). The former is found in the soluble fraction and migrates faster in SDS–PAGE, whereas the latter is found in membrane pellets upon carbonate extraction and migrates more slowly (Fig. S1A). These data suggest that SS cleavage may differ depending on the length of the N terminus preceding the SS (N-length); thus, we attempted to determine the relationship between the N-length and the efficiency of SS cleavage by systematic truncation of the N terminus of N26CPY.

**Fig. 1.**
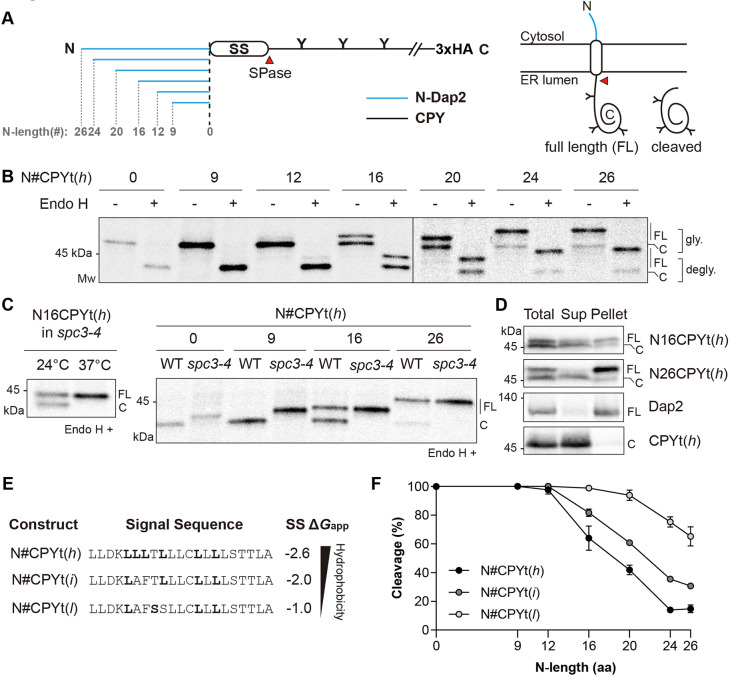
**Signal sequence processing by SPase depends on the *n* region length and *h* region hydrophobicity of SSs.** (A) Schematic of N#CPYt constructs. Left: blue lines indicate N-terminal extensions derived from the N terminus of the yeast membrane protein Dap2 (N-Dap2), and the black line indicates the yeast vacuole protein CPY. Numbers indicate the extended amino acids. N-linked glycosylation sites are shown as ‘Y’. HA, hemagglutinin tag. Right: diagram showing possible forms of N#CPYt variants in the ER. (B) Yeast transformants carrying the indicated N#CPYt(*h*) constructs were radiolabeled for 5 min at 30°C, immunoprecipitated using an anti-HA antibody, subjected to SDS–PAGE and analyzed by autoradiography. Endo H treatment was performed prior to SDS–PAGE. FL, full length; C, cleaved; gly., glycosylated species; degly., deglycosylated species. (C) Left: *spc3-4* cells expressing N16CPYt(*h*) were incubated and radiolabeled for 5 min at the indicated temperatures. Right: the indicated N#CPYt(*h*) variants in the WT or *spc3-4* strain were analyzed as in B, except that N#CPYt(*h*) variants in the *spc3-4* strain were incubated at 37°C for 30 min prior to radiolabeling and were radiolabeled at 37°C. All the samples were treated with Endo H prior to SDS–PAGE. (D) The indicated CPY variants and Dap2 were subjected to carbonate extraction, and the resulting protein samples were detected by western blotting using an anti-HA antibody (Sup, supernatant). (E) Hydrophobicities of the N#CPYt variant SSs were predicted using the Δ*G* predictor (Δ*G*_app_ in kcal/mol; http://dgpred.cbr.su.se/index.php?p=home). Amino acids shown in bold indicate the modified residues from the SS of WT CPY. (F) The relative amounts of SPase-processed species over glycosylated products for each CPY variant were measured and plotted (as percentage cleavage). The *x*-axis indicates the number of amino acids (aa) preceding the SS (N-length). At least three independent experiments were carried out. Data are presented as mean±s.d. Data in B are representative of three independent experiments. Data in C and D are representative of two independent experiments.

For better separation of the size of cleaved and uncleaved species on SDS–PAGE, the C terminus was shortened to residue 323 of CPY [N#CPYt(*h*), where t denotes the C-terminal truncation and N# denotes the number of amino acids in the N-terminal extension] (Fig. 1A, Table 1). To capture the early stage of protein translocation and processing, yeast cells carrying N#CPYt(*h*) variants were radiolabeled with [^35^S]-Met for 5 min. Radiolabeled proteins were immunoprecipitated using anti-HA antibodies directed to the HA epitope at their C terminus, subjected to SDS–PAGE and analyzed by autoradiography. Proper targeting and translocation of CPY to the ER was determined by assessing the glycosylation status of CPY, as the protein contains three N-linked glycosylation sites, which are glycosylated in the ER lumen. All N#CPYt(*h*) variants were sensitive to treatment with endoglycosidase H (Endo H), which removes N-linked glycans, indicating that they were efficiently translocated into the lumen (Fig. 1B).

**Table 1.**
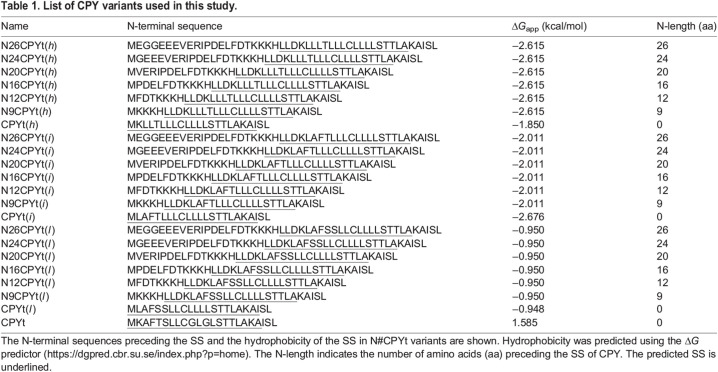
List of CPY variants used in this study.

Two bands were detected for the longer N-length variants [N16CPYt(*h*) to N26CPYt(*h*)] even after Endo H treatment, indicating that they represent proteins of two different sizes in the ER (Fig. 1B). When the sizes of CPYt(*h*) [N#CPYt(*h*) with no N-terminal extension] and N16CPYt(*h*) were compared to that of CPYt(wt) (CPYt possessing the original SS), CPYt(*h*) and the smaller size form of N16CPYt(*h*) migrated the same as CPYt(wt), the SS of which is efficiently cleaved by SPase. Thus, CPYt(*h*) is fully cleaved and N16CPYt(*h*) is partially cleaved by SPase (Fig. S1B). We also prepared an N-terminal SS-deleted version of CPYt(*h*) (mCPYt), which was expressed *in vitro*, and compared its size with the Endo H-treated sample of N16CPYt(*h*) expressed *in vivo*. A fast-migrating, deglycosylated band of N16CPYt(*h*) and an *in vitro*-translated product of mCPYt were resolved at the same size on an SDS–PAGE gel, confirming that the former is an SS-cleaved CPY (Fig. S1C).

To further confirm the SPase-mediated cleavage, selected N#CPYt(*h*) variants were expressed in the *spc3-4* yeast strain, which exhibits a temperature-sensitive defect in SPase activity (Fang et al., 1997). When cells carrying N16CPYt(*h*) were radiolabeled at a permissive temperature of 24°C, two forms of the protein were observed as separate bands, whereas the lower band was not observed in cells radiolabeled at the nonpermissive temperature of 37°C, indicating that the lower band resulted from SPase activity (Fig. 1C). CPYt(*h*) and N9CPYt(*h*) variants expressed at 37°C in the *spc3-4* strain migrated slower than those expressed in the wild-type (WT) strain, and fast-migrating bands of the N16CPYt(*h*) and N26CPYt(*h*) variants in the *spc3-4* strain were no longer detected when cells expressing these variants were labeled at 37°C (Fig. 1C).

Finally, we determined the localization of SS-cleaved and -uncleaved species using carbonate extraction followed by western blotting. SS-cleaved forms of N16CPYt(*h*) and N26CPYt(*h*) variants were found in soluble fractions, while the uncleaved forms were mainly found in pellet fractions, indicating that the latter became membrane anchored (Fig. 1D).

These data show that SSs of N#CPYt(*h*) variants with shorter N-lengths [CPYt(*h*), N9CPYt(*h*) and N12CPYt(*h*)] were efficiently cleaved, whereas cleavage gradually decreased for variants with longer N-lengths, indicating that SSs with shorter N-lengths are better substrates for SPase than SSs with longer N-lengths.

We next investigated the effect of the hydrophobicity of the SS on cleavage by SPase. Sets of N#CPYt variants having SSs of intermediate hydrophobicity [N#CPYt(*i*); *i* for intermediate hydrophobicity] and low hydrophobicity [N#CPYt(*l*); *l* for low hydrophobicity] were prepared and analyzed by 5 min pulse labeling, as above (Fig. 1E; Fig. S1D). The relative amounts of SS-cleaved species among the glycosylated products were quantified (expressed as percentage cleavage; Fig. 1F). The SS cleavage profiles of the N#CPYt(*i*) and N#CPYt(*l*) variants were shifted to the right compared to that of N#CPYt(*h*) (Fig. 1F). These data show that the N-length and hydrophobicity of SSs are two critical determinants based on which SPase selects substrates.

### Internal SSs are more efficiently cleaved by SPase lacking Spc1

**Fig. 2.**
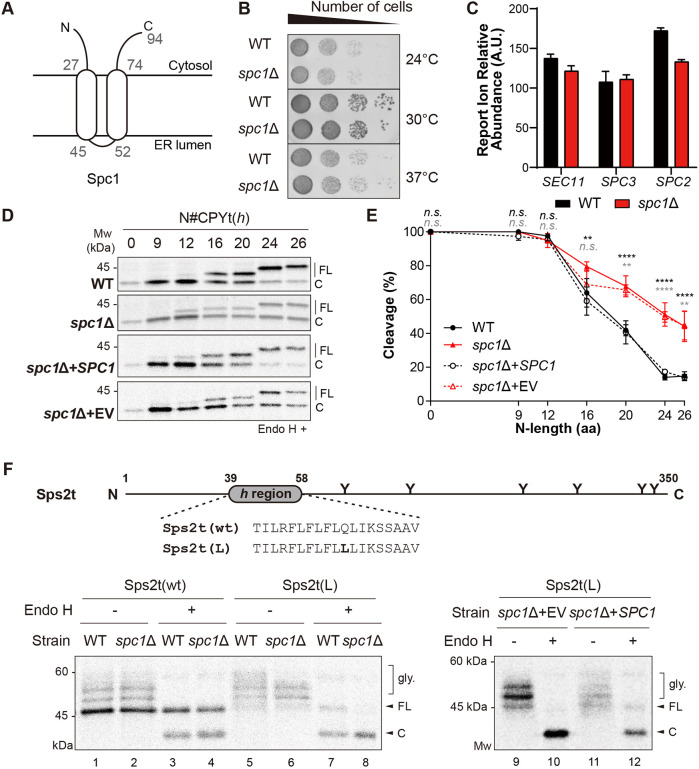
**Cleavage of internal SSs is enhanced in the absence of Spc1.** (A) Schematics of the membrane topology of Spc1. Numbers indicate amino acid positions and highlight the ends of two TM domains of Spc1. (B) WT and *spc1*Δ cells were serially diluted from 0.2 OD_600_ cells and grown on YPD medium for 1 day at the indicated temperatures. Images are representative of three independent experiments. (C) The abundance of other SPC subunits, Sec11, Spc3 and Spc2, in the WT and *spc1*Δ strains was assessed using mass spectrometry. The mean±s.d. of three repeats is shown. (D) N#CPYt(*h*) variants in the WT, *spc1*Δ, *spc1*Δ+*SPC1* and *spc1*Δ+EV strains were assessed as in Fig. 1B. All the samples were treated with Endo H prior to SDS–PAGE. (E) Percentage cleavage of N#CPYt(*h*) variants in the indicated strains was analyzed. At least three independent experiments were carried out. Data are presented as mean±s.d. *P*-values between WT and *spc1*Δ and between *spc1*Δ+EV and *spc1*Δ+*SPC1* were calculated by multiple two-tailed, unpaired Student's *t*-tests and are shown in black and gray colors, respectively (n.s., *P*>0.05; **P≤0.01; ****P≤0.0001). (F) Top: schematic of Sps2t variants. Numbers indicate amino acid positions, glycosylation sites are shown as ‘Y’. Leucine mutation is shown in bold. Bottom: processing of Sps2t variants in WT, *spc1*Δ, *spc1*Δ+*SPC1* and *spc1*Δ+EV strains. Experimental procedures were carried out as in Fig. 1B. Data are representative of three independent experiments. aa, amino acids; A.U., arbitrary units; C, cleaved; FL, full length; gly., glycosylated species.

The eukaryotic SPase has multiple subunits, and the functions of each subunit remain poorly defined. We set out to investigate the role of Spc1, a small, 94-residue-long membrane protein subunit (Fang et al., 1996; Kalies and Hartmann, 1996). Spc1 spans the ER membrane twice, with both termini facing the cytoplasm and a very short loop in the lumen (Fig. 2A).

First, we prepared an *spc1*Δ strain and assessed its growth phenotype. No growth defect was observed at all tested temperatures (Fig. 2B), as seen in a previously published study (Fang et al., 1996). To check whether the deletion of Spc1 affects the stability of the other subunits of SPase, we carried out mass spectrometry analysis to compare the abundance of Sec11, Spc3 and Spc2 in WT and *spc1*Δ cells. Although the abundance of the nonessential subunit Spc2 was slightly reduced in the *spc1*Δ strain, the abundance of Sec11 and Spc3, which are the catalytic components of SPase, was unchanged (Fig. 2C), indicating that Spc1 deletion hardly affects the stability of other subunits in the complex. Furthermore, SS cleavage of shorter N#CPYt variants in *spc1*Δ cells occurred efficiently, indicating that SPase activity is not impaired in the absence of Spc1 (Fig. 2D,E; Fig. S2).

For N#CPYt(*h*) variants with N-lengths longer than 16 amino acids, SS cleavage was increased in *spc1*Δ cells compared to that in WT cells (Fig. 2D,E). The difference between the SS cleavage efficiency in *spc1*Δ and WT cells became larger as the N-length became longer (Fig. 2D,E). SS cleavage was also assessed in an *spc1*Δ strain carrying either a plasmid with *SPC1* under the control of its own promoter or an empty vector (EV). Cleavage efficiencies of N#CPYt(*h*) variants in an *spc1*Δ strain with *SPC1* were restored to the level observed in the WT strain, confirming that increased cleavage of longer N-length variants in the *spc1*Δ strain was due to the absence of Spc1 (Fig. 2D,E).

Less hydrophobic N#CPYt(*i*) and N#CPYt(*l*) variant sets showed similar cleavage patterns: cleavage efficiency increased for the longer N-length variants in the *spc1*Δ strain and was restored when *SPC1* was added (Fig. S2A,B). These data suggest that membrane-anchored sequences are more readily cleaved when Spc1 is absent.

### Processing of Sps2

Sps2 is a protein involved in sporulation and is localized to the plasma membrane and cell wall in *S. cerevisiae* (Coluccio et al., 2004). It contains an internal SS that some prediction programs predict to be a putative TM. We prepared a truncated form of Sps2 [Sps2t(wt)] containing the N-terminal 350 residues (full-length Sps2 is 502 residues long) to facilitate the separation of SS-cleaved and -uncleaved forms on SDS–PAGE gels (Fig. 2F). After 5 min of radiolabeling, the unglycosylated protein was detected in WT and *spc1*Δ strains, indicating inefficient targeting to the ER (Fig. 2F). Since inefficient ER targeting obscures analysis of cleavage, a single amino acid substitution was made in the *h* region to create a truncated Sps2 variant with improved ER targeting [Sps2t(L)] (Fig. 2F). Because untargeted proteins accumulated at 37°C, we assessed the cleavage at 33°C, a semipermissive temperature, without compromising ER targeting and confirmed that Sps2t(L) is cleaved by SPase (Fig. S2C). Although a full-length form was detected, the majority of Sps2t(L) was processed by SPase in the WT strain, as assessed using 5 min pulse labeling, indicating that Sps2 has an internal cleavable SS. In the *spc1*Δ strain, no full-length Sps2t(L) product was detected following 5 min pulse labeling (Fig. 2F), suggesting that cleavage of the internal SS of Sps2 occurs faster in the absence of Spc1.

### Recognition and usage of the SS cleavage site by SPase is unchanged without Spc1

**Fig. 3.**
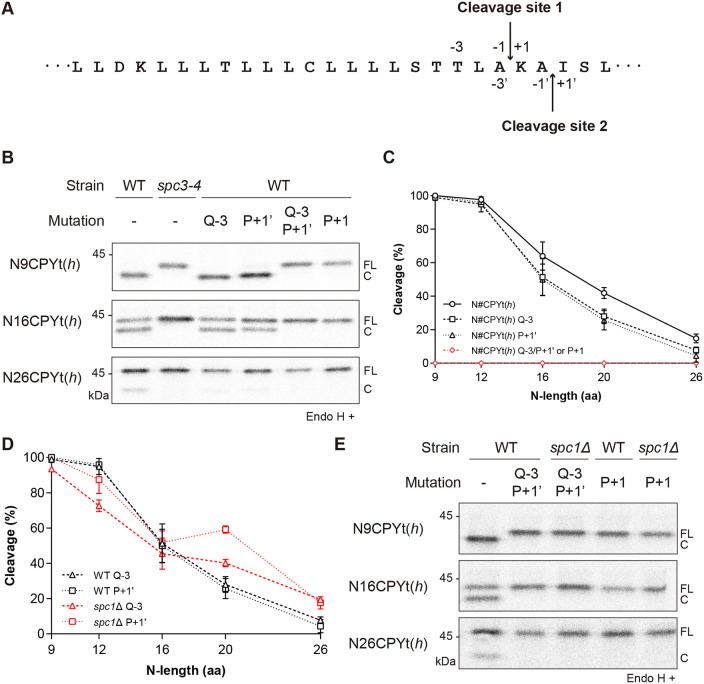
**Recognition and usage of the SS cleavage site by SPase is unchanged in the *spc1*Δ strain.** (A) Two cleavage sites are present in the SS of N#CPYt(*h*): cleavage site 1 and cleavage site 2 are indicated as downward and upward arrows, respectively. (B) The indicated cleavage site mutants of N#CPYt(*h*) variants in the WT or *spc3-4* strain were radiolabeled for 5 min at 30°C (37°C for *spc3-4*), immunoprecipitated using anti-HA antibodies, subjected to SDS–PAGE and Endo H treatment, and analyzed by autoradiography. (C) Percentage cleavage of the cleavage site mutants in B was analyzed as in Fig. 1F and compared. At least three independent experiments were carried out. Data are presented as mean±s.d. (D) Percentage cleavage of N#CPYt(*h*) variants with Q−3 or P+1′ mutations in the WT or *spc1*Δ strain is compared. At least three independent experiments were carried out. Data are presented as mean±s.d. (E) The indicated N#CPYt(*h*) variants lacking canonical cleavage sites in the WT or *spc1*Δ strain were radiolabeled and assayed as described in B. Data are representative of two independent experiments. aa, amino acids; C, cleaved; FL, full length.

We wondered whether the expanded substrate range of SPase in *spc1*Δ cells is due to altered recognition and usage of cleavage sites by SPase lacking Spc1 and investigated the cleavage sites of CPY variants in WT and *spc1*Δ cells. When the SS cleavage sites of CPY variants were searched using SignalP-5.0 (http://www.cbs.dtu.dk/services/SignalP/; Almagro Armenteros et al., 2019), two sites were predicted for all CPYt variants (including wild-type CPY) with equal probabilities (0.492 for the upstream cleavage site and 0.482 for the downstream cleavage site; Fig. 3A and Fig. S3A). Hereafter, we refer to the upstream and downstream cleavage sites as cleavage sites 1 and 2, respectively, and denote residue positions relative to cleavage site 2 using prime symbols (e.g. −3′, −1′; Fig. 3A).

To identify which cleavage site is used, cleavage site 1 and 2 were selectively eliminated individually by single amino acid substitutions in N#CPYt(*h*) variants. Given that the canonical SS cleavage sites follow the ‘−3, −1 rule’ and that proline (P) at the +1 position with respect to the cleavage site inhibits SS processing (Cui et al., 2015; Nilsson and von Heijne, 1992; Barkocy-Gallagher and Bassford, 1992), a residue at the −3 position in cleavage site 1 was replaced with the polar and bulky residue glutamine (Q−3), and the +1′ position in cleavage site 2 was replaced with proline (P+1′) (Fig. 3B). Predictions by SignalP-5.0 showed a single cleavage site for each mutant, indicating that Q−3 and P+1′ substitutions disrupt cleavage sites 1 and 2, respectively (Fig. S3B,C). To confirm that cleavage site 1 or 2 were selectively eliminated by Q−3 or P+1′ substitution, N#CPYt(*h*) variants carrying the double mutation Q−3/P+1′ were also prepared.

Cleavage of N#CPYt(*h*) variants possessing cleavage site mutations was assessed using 5 min pulse labeling experiments, and the data were compared with the cleavage profile of N#CPYt(*h*) variants. Although a slightly decreased cleavage of N16CPYt(*h*), N20CPYt(*h*) and N26CPYt(*h*) was observed upon inhibition at cleavage site 1 (Q−3) or site 2 (P+1′), the overall pattern of the cleavage profile remained the same regardless of whether the variants contained both cleavage sites or only cleavage site 1 or 2, suggesting that SPase recognizes and uses both sites efficiently (Fig. 3B,C). On the other hand, the double mutation Q−3/P+1′ completely abolished SS processing of all variants. When proline was introduced at the +1 position for cleavage site 1 (P+1), which is the −2′ position for cleavage site 2, SS cleavage was also completely blocked, indicating that both sites were inhibited by the presence of proline at this position (Fig. 3B,C). We wondered whether N9CPYt(*h*), a short N-length variant, was present in the membrane when uncleaved, and carried out carbonate extraction to assess this. N9CPYt(*h*) with the Q−3/P+1′ mutation was found in the membrane pellet, showing that the protein becomes membrane-anchored when unprocessed by SPase (Fig. S3D).

To determine whether SPase lacking Spc1 uses different cleavage sites for processing, we analyzed SS processing of the cleavage site variants in *spc1*Δ cells. Cleavage of N20CPYt(*h*) and N26CPYt(*h*) variants with Q−3 or P+1′ mutations in *spc1*Δ cells increased compared to that in WT cells (Fig. 3D). These results indicate that recognition and usage of cleavage sites are unchanged for SPase without Spc1. Next, we set out to determine whether SPase lacking Spc1 uses a noncanonical SS cleavage site, thereby evading the −3, −1 rule for processing. Three N#CPYt(*h*) variants with the Q−3/P+1′ or P+1 mutation that eliminated both canonical SS cleavage sites were expressed in *spc1*Δ cells, and their cleavage was assessed (Fig. 3E). As in WT cells, no cleavage was detected for these sets of variants, indicating that SPase still processes the canonical SS cleavage sites, even in the absence of Spc1 (Fig. 3E).

### SPase-mediated cleavage of TM segments in membrane proteins is enhanced in the absence of Spc1

Having observed that SPase lacking Spc1 processes internal, membrane-anchored SSs, we reasoned that TM segments of membrane proteins may also be subjected to SPase-mediated cleavage in *spc1*Δ cells. To test this idea, LepCC membrane proteins, derived from *E. coli* leader peptidase (Lep), were used. LepCC proteins contain an engineered TM2 segment composed of Leu residues followed by a cleavage cassette (VPSAQA↓A, where ↓ indicates the cleavage site of SPase; Fig. S4A) (Nilsson et al., 1994). A previous study has shown that SPase-mediated cleavage after TM2 of these proteins is dependent on the number of Leu residues in TM2, as determined *in vitro* using dog pancreatic microsomes; TM2 variants with a shorter stretch of Leu residues are cleaved by SPase, whereas TM2 variants with a longer stretch of Leu residues remain uncleaved (Nilsson et al., 1994).

**Fig. 4.**
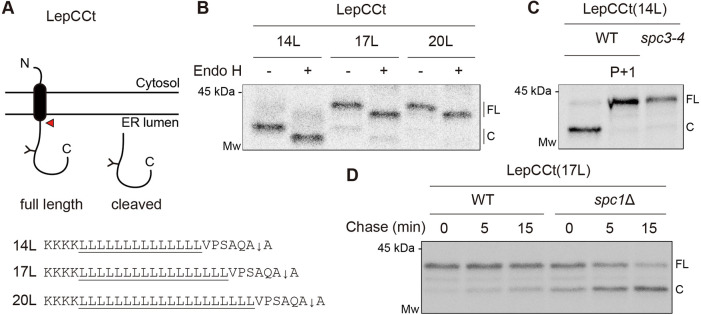
**SPase-mediated processing of signal-anchored proteins is enhanced in the *spc1*Δ strain.** (A) Schematic of LepCCt. The TM domain is colored black, and an N-linked glycosylation site is indicated as ‘Y’. A red arrowhead points to the site of cleavage by SPase. Flanking and TM sequences including the cleavage site (downwards arrow) are shown for three LepCCt variants. Leucine repeats are underlined. (B) The indicated LepCCt variants in WT cells were radiolabeled for 5 min at 30°C and subjected to immunoprecipitation for SDS–PAGE and autoradiography. Protein samples were treated with or without Endo H prior to SDS–PAGE. (C) The LepCCt(14L) construct in the WT or *spc3-4* strain was radiolabeled and analyzed as in B. P+1 in LepCCt(14L) indicates proline substitution in the +1 position relative to the cleavage site. (D) LepCCt(17L) in the WT or *spc1*Δ strain was radiolabeled for 5 min and chased for the indicated time points at 30°C, then immunoprecipitated with anti-HA antibodies, subjected to SDS–PAGE and analyzed by autoradiography. Data in B are representative of three independent experiments. Data in C and D are representative of two independent experiments. C, cleaved species; FL, full length.

We deleted the N terminus, including the first TM, of LepCC to generate signal-anchored LepCC versions with 14 leucines [LepCCt(14L)], 17 leucines [LepCCt(17L)], and 20 leucines [LepCCt(20L)] in their TMs and subcloned the fragments in a yeast expression vector (LepCCt; Fig. 4A). All three constructs were expressed in yeast cells. Upon Endo H treatment, the LepCCt band shifted down for all LepCCt variants, indicating efficient translocation and membrane insertion in the yeast ER (Fig. 4B). For LepCCt(14L), the size of the major band was smaller than the expected full-length protein (Fig. 4B). To determine whether smaller band size resulted from SPase-mediated processing, we adopted two strategies. First, an SS cleavage site was destroyed by introducing proline at the +1 position in LepCCt(14L); and second, LepCCt(14L) was radiolabeled in the *spc3-4* strain at the nonpermissive temperature (Fig. 4C). A slowly migrating full-length band was detected for LepCCt(14L) with a cleavage site mutation (P+1) or when expressed in the *spc3-4* strain at the nonpermissive temperature, whereas a fast-migrating product was predominant when expressed in the WT strain, confirming that LepCCt(14L) was processed by SPase in yeast. Albeit less prominently, LepCCt(17L) also generated a fast-migrating band, indicating that it is also a substrate for SPase (Fig. 4B).

We next traced processing of the LepCCt(17L) variant in the WT and *spc1*Δ strains using pulse-chase experiments (Fig. 4D). The abundance of a cleaved product of LepCCt(17L) in the *spc1*Δ strain significantly increased at 0 min compared to that expressed in the WT strain and further increased in the following chase time, indicating that SPase-mediated cleavage continued post-translationally (Fig. 4D). We also determined cleavage of double-spanning LepCC variants in WT and *spc1*Δ cells and observed that SPase-mediated cleavage of a TM domain increased in *spc1*Δ cells, similar to the cleavage of single-spanning LepCCt variants (Fig. S4A,B). These data suggest that longer TM segments normally evade SPase-mediated processing, but they are subjected to cleavage by SPase when Spc1 is absent.

**Fig. 5.**
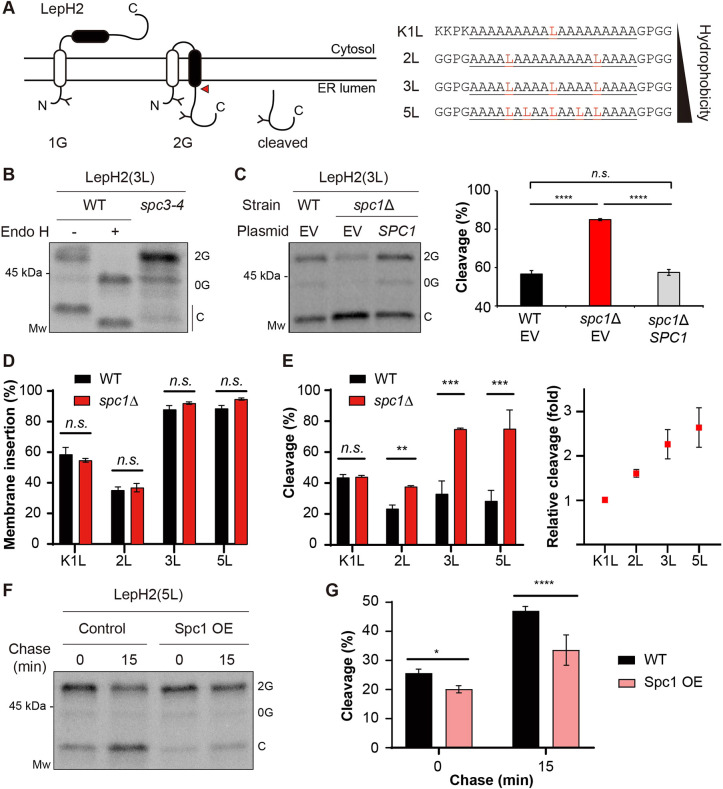
**SPase-mediated processing of double-spanning membrane proteins is modulated by Spc1.** (A) Schematics of LepH2. The second hydrophobic segment (H) of varying hydrophobicity is colored black. N-linked glycosylation sites are indicated as ‘Y’, and a red arrowhead points to the site of cleavage by SPase. 1G, singly glycosylated form; 2G, doubly glycosylated form. Amino acid sequences of the H segment are shown and underlined, with N- and C-terminal flanking residues. Leucine residues are highlighted. (B) LepH2(3L) in the WT or *spc3-4* strain was radiolabeled for 5 min at 30°C. WT samples were treated with Endo H prior to SDS–PAGE. 0G, nonglycosylated form; 2G, doubly glycosylated form; C, cleaved form after membrane insertion. (C) LepH2(3L) in the WT+EV, *spc1*Δ+EV or *spc1*Δ+*SPC1* strain was radiolabeled for 5 min at 30°C and analyzed. Left: autoradiogram of a representative blot is shown. Right: cleavage of the LepH2 H segments was calculated as a percentage of the cleaved band intensity over the sum of the 2G and cleaved band intensities from three independent experimental measurements. (D) Percentage membrane insertion of the H segment in LepH2 variants was measured as 2G/(total−0G)×100. (E) Left: percentage cleavage of the LepH2 H segments was calculated as in C. Right: relative cleavage of the LepH2 H segments in *spc1*Δ cells compared to that in WT cells was calculated as the ratio of percentage cleavage in *spc1*Δ cells and WT cells, and is plotted for each LepH2 variant. (F) LepH2(5L) in WT cells harboring control vector or Spc1 overexpression (OE) vector. Transformants were subjected to radiolabeling for 5 min at 30°C followed by a chase for the indicated time points. (G) Percentage cleavage of LepH2(5L) in F was calculated as in C. For all the experimental sets, at least three independent experiments were carried out, and data are presented as mean±s.d. *P≤0.05; **P≤0.01; ***P≤0.001; ****P≤0.0001; n.s., *P*>0.05 (two-tailed, unpaired Student's *t*-tests).

Next, to determine the effect of TM segment hydrophobicity on SPase-mediated cleavage, we tested another set of *E. coli* Lep-derived membrane proteins harboring an engineered TM2 composed of Leu and Ala residues with a fixed length of 19 residues (LepH2; Fig. 5A) (Lundin et al., 2008). The TM2 segment becomes more hydrophobic with an increasing number of Leu residues. Previously, it was shown that LepH2 variants undergo SPase-mediated cleavage both *in vitro* in dog pancreas microsomes and *in vivo* in yeast cells (Lundin et al., 2008). We confirmed that the cleaved fragment was generated by SPase by expressing a LepH2 variant with three Leu residues [LepH2(3L)] in the *spc3-4* strain at the nonpermissive temperature (Fig. 5B).

LepH2(3L) was expressed in WT and *spc1*Δ strains carrying either an EV or a plasmid bearing the *SPC1* gene to assess whether re-expression of Spc1 restores LepH2(3L) processing, as in the WT strain. Indeed, cleavage of LepH2(3L) in the *spc1*Δ strain with *SPC1* was comparable to that in the WT strain with an EV, reaching ∼55%, whereas cleavage of LepH2(3L) in the *spc1*Δ strain with an EV resulted in ∼85% cleavage. These data show that Spc1 regulates SPase processing of LepH2 (Fig. 5C).

Additional LepH2 variants of varying hydrophobicities were expressed in WT and *spc1*Δ strains and analyzed. Membrane insertion of TM2 of LepH2 variants in WT and *spc1*Δ cells remained unchanged, demonstrating that deletion of Spc1 does not interfere with membrane insertion of TM2 (Fig. 5D). However, cleavage of LepH2 constructs in the *spc1*Δ strain increased in a hydrophobicity-dependent manner; cleavage of hydrophobic LepH2(3L) and LepH2(5L) was significantly increased in the *spc1*Δ strain (>2-fold; Fig. 5E). LepH2(K1L) contains positively charged N-terminal flanking residues that enhance membrane insertion of TM2 despite low hydrophobicity. Although membrane insertion of LepH2(K1L) was increased relative to that of LepH2(2L), cleavage of LepH2(K1L) in *spc1*Δ cells did not increase, whereas cleavage of LepH2(2L) did. These data collectively suggest that TM segment hydrophobicity may be an important determinant for Spc1-regulated SPase processing of membrane proteins (Fig. 5D,E).

### SPase-mediated cleavage of TM segments is decreased upon overexpression of Spc1

Since processing of test membrane proteins by SPase increased in the absence of Spc1, we wondered whether overexpression of Spc1 also affects processing, possibly in an opposite manner. The LepH2(5L) construct was radiolabeled then chased for 15 min in the WT strain containing an EV or Spc1 overexpression (OE) vector (Fig. 5F). Cleavage of LepH2(5L) in both strains was increased during the 15 min chase, indicating that cleavage continued post-translationally. At 0 min chase, cleavage of LepH2(5L) in the Spc1 OE cells was slightly reduced compared to the cleavage in WT cells, but at 15 min chase, cleavage in Spc1 OE cells was markedly reduced compared to that in WT cells (Fig. 5F,G). Under Spc1 OE conditions, cleavage of other test membrane proteins, the double-spanning LepCC(17L) and single-spanning LepCCt(17L) variants, also decreased compared to cleavage of those expressed in WT cells (Fig. S4C,D). These data suggest that additional Spc1 can protect TM segments from SPase-mediated cleavage co- and post-translationally.

### Overexpressed Spc1 interacts with membrane proteins with uncleaved TM segments

**Fig. 6.**
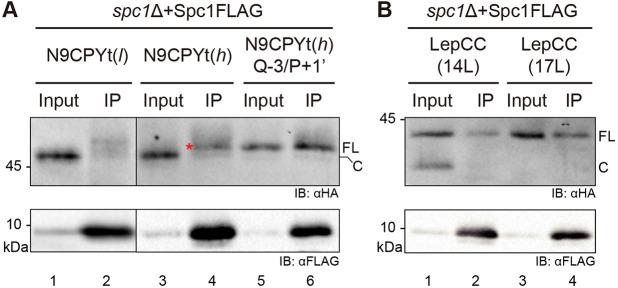
**Overexpressed Spc1 interacts with model membrane proteins.** Co-immunoprecipitation of overexpressed FLAG-tagged Spc1 with (A) N9CPYt and (B) LepCC variants. *spc1*Δ cells co-expressing Spc1–FLAG and the indicated HA-tagged substrates were subjected to crude membrane fractionation. Isolated membranes were solubilized with 1% Triton X-100 lysis buffer, followed by co-immunoprecipitation with anti-FLAG antibodies and visualization using SDS–PAGE and immunoblotting (IB) with the indicated antibodies. Representative blots from three experiments are shown. IP; immunoprecipitates; FL, full-length; C, cleaved. A red asterisk indicates full-length N9CPYt. Input lanes represent 2% of the total lysate.

We wondered whether overexpressed Spc1 interacts with membrane proteins, thereby protecting TMs from SPase-mediated processing, and carried out co-immunoprecipitation to assess this (Fig. 6). We used three N9CPYt variants; two variants carrying the cleavable SS, N9CPYt(*l*) and N9CPYt(*h*), and an N9CPYt(*h*) Q−3/P+1′ version carrying the uncleavable SS, which acts as a membrane anchor (Fig. S3D). Although N9CPYt(*h*) was fully cleaved in the WT and *spc1*Δ strains (Fig. 2D), we observed that some uncleaved N9CPYt(*h*) was selectively pulled down with overexpressed Spc1 (Fig. 6A, lane 4). Since uncleaved N9CPYt(*h*) was not detected in the input sample (Fig. 6A, lane 3), this must be a minor fraction protected from SPase cleavage by associating with overexpressed Spc1. These data suggest that overexpressed Spc1 reduces cleavage of N9CPYt(*h*), as observed with other model membrane proteins above. N9CPYt(*l*), a less hydrophobic version of N9CPYt(*h*), was not co-immunoprecipitated with overexpressed Spc1, whereas N9CPYt(*h*) Q−3/P+1′ was (Fig. 6A, lanes 2 and 6, respectively). Although both N9CPYt(*h*) variants with and without cleavage site were co-immunoprecipitated with overexpressed Spc1, the less hydrophobic N9CPYt(*l*) was not, suggesting that Spc1 recognizes a hydrophobic segment rather than the cleavage site.

Next, we tested LepCC(14L) and LepCC(17L) variants. While a cleaved product of LepCC(14L) was not co-immunoprecipitated with Spc1, a full-length protein was (Fig. 6B, lane 2). A full-length LepCC(17L) was also co-precipitated with Spc1 (Fig. 6B, lane 4). These data suggest that Spc1 interacts with membrane proteins, in line with previous studies showing that SPCS1, a human homolog of Spc1, interacts with the TMs of viral proteins (Ma et al., 2018; Suzuki et al., 2013).

## DISCUSSION

The sequence contexts of SPs that are cleaved by SPase and of signal-anchored segments that become TM domains are similar in their hydrophobicity and overall length. Hence, both can be recognized by the signal recognition particle (SRP), act as an ER targeting signal and initiate protein translocation in the ER membrane. In a subsequent step, an SP is cleaved, whereas a TM segment evades processing by SPase and anchors in the membrane. Although the cleavage motif is needed, it is not the sole factor that determines whether SPase cleaves the signal sequence or not. It remains elusive how SPs and TMs are sorted by SPase.

Analyzing the cleavage of CPY-based SSs of systematically varied N-length and hydrophobicity, our data show that the substrate spectrum of SPase is defined by the N-length and hydrophobicity of SSs; SSs with shorter *n* regions and/or less hydrophobic *h* regions are better substrates for SPase. Consistent with our findings, it has been observed that some type II single-pass membrane proteins are processed by SPase when their *n* region is shortened in mammalian cells (Lemire et al., 1997; Lipp and Dobberstein, 1986; Roy et al., 1993; Schmid and Spiess, 1988).

When the cleavage of the same CPY variants was assessed in the absence of Spc1, cleavage of internal signal-anchored sequences was markedly enhanced, and the cleavage pattern was restored when *SPC1* was re-expressed. There are two possible explanations for the expanded CPY substrate spectrum of SPase in the absence of Spc1: (1) SPase may cleave SSs in sites other than the canonical site or (2) SPase may cleave signal peptide-like sequences such as TM domains due to compromised capacity for substrate selection. We carried out mutational analysis of the SS cleavage sites of CPY variants to test the first possibility and found that SPase only processes the canonical SS cleavage site with or without Spc1, excluding the first possibility. For the second possibility, we tested the processing of membrane proteins in the *spc1*Δ strain. A pulse-chase experiment showed that cleavage of TM segments in model membrane proteins in *spc1*Δ cells was enhanced at early time points of metabolic labeling and further increased at subsequent chase times, indicating that processing continues after membrane insertion. We also observed that SPase-mediated cleavage of TM segments of model membrane proteins was reduced upon overexpression of Spc1. These results suggest that the expanded CPY and Lep membrane protein substrate spectrum of SPase without Spc1 is due to a compromised capability to exclude TM segments from SPase action.

How does Spc1 sort out TM segments and exclude them from SPase action? Our co-immunoprecipitation data show that Spc1 interacts with membrane proteins with uncleaved TM segments. These results suggest that Spc1 may shield TM segments from being presented to the active site of SPase, thereby protecting them.

Although Spc1 is dispensable for growth in yeast, deletion of SPC12 (an Spc1 homolog) in *Drosophila* causes a developmental-lethal phenotype (Haase Gilbert et al., 2013), suggesting that its function may be more prominent in higher eukaryotes. Intriguing observations have been made in SPCS1-knockout human cell lines. A genetic screen has identified SPCS1 as one of the key regulators of the expression of ULBP1, a surface protein ligand for natural killer cells (Gowen et al., 2015). Genome-wide CRISPR screening has identified SPCS1 as a key host factor in the processing of viral proteins that are made as polyproteins containing internal SSs and TM segments during infection by viruses of the flavivirus family (Zhang et al., 2016). Interestingly, host SPCS1 has been found to interact with the TM domains of viral proteins in Japanese encephalitis virus (Ma et al., 2018) and with the TM domains of viral proteins in hepatitis C virus (Suzuki et al., 2013). These observations indicate that SPCS1 is involved in regulating the processing and handling of TM segments in higher eukaryotes.

Our study provides evidence that SPase distinguishes SPs from TM segments and that Spc1 is involved in deselecting TM segments, thereby sharpening SPase substrate selection and acting as a negative regulator of the SPase-mediated processing of membrane proteins.

## MATERIALS AND METHODS

### Yeast strains

The *S. cerevisiae* haploid W303-1α (*MATα*, *ade2*, *can1*, *his3*, *leu2*, *trp1*, *ura3*) was used as the WT strain. The *SPC1* ORF in W303-1α was replaced with *HIS3* amplified from the pCgH plasmid (Kitada et al., 1995) by homologous recombination to generate the *spc1*Δ strain (*MATα*, *spc1Δ::HIS3*, *ade2*, *can1*, *his3*, *leu2*, *trp1*, *ura3*). *spc3-4* is a temperature-sensitive mutant exhibiting a defect in SPase activity at 37°C (Fang et al., 1997). For the overexpression of Spc1–FLAG, the pRS426 vector (Christianson et al., 1992) carrying *SPC1–FLAG* under the GPD promoter was transformed into the W303-1α strain.

### Construction of plasmids

All CPY variants were derived from pRS424GPD N26CPY-HA, which was constructed in our previous study (Yim et al., 2018). Using this construct as a template, we first truncated residues 323–532 of CPY by site-directed mutagenesis following the manufacturer's protocol (KOD-Plus-Mutagenesis Kit; Toyobo, Japan). Next, the N terminus was truncated, and the hydrophobicity of the CPY SS was modified by site-directed mutagenesis. *E. coli* Lep-derived LepCC constructs (Nilsson et al., 1994) were subcloned from the pGEM4z vector into the yeast pRS424 vector by PCR amplification and homologous recombination. LepCCt constructs were generated by truncation of the N-terminal 20 residues, except for the start methionine, in pRS424GPD LepCC constructs. The pRS426 vector containing *SPC1* was cloned by homologous recombination or using the Gibson assembly protocol. A 2 μl volume of PCR fragments of a target gene, 0.5 μl of restriction enzyme-linearized vector and 2.5 μl of 2× Gibson mix [167 mM Tris-HCl, pH 7.5, 16.7 mM MgCl_2_, 0.3 mM dATP, 0.3 mM dCTP, 0.3 mM dGTP, 0.3 mM dTTP, 16.7 mM dithiothreitol (DTT), 8.3% PEG-8000, 1.67 mM oxidized nicotinamide adenine dinucleotide, 0.008 U T5 exonuclease (NEB, M0363S), 0.05 U Phusion polymerase (Thermo Fisher Scientific) and 8 U Taq ligase (NEB, M0208S)] were mixed, incubated at 50°C for 1 h and transformed into *E. coli*. Single colonies were picked, and resulting plasmids were sequenced to select appropriate clones. All plasmids were confirmed by DNA sequencing. The LepH2 variants in a yeast vector were constructed and described previously (Lundin et al., 2008).

### Pulse labeling and pulse-chase experiments

Pulse labeling and pulse-chase procedures were carried out as described previously (Reithinger et al., 2014; Yim et al., 2018). Briefly, yeast cells were grown at 24–30°C until the OD_600_ reached 0.3–0.8 in selective medium. Then, 1.5 OD_600_ units of cells were harvested by centrifugation (2170 ***g***, 5 min, 4°C), washed with −Met medium without ammonium sulfate, and incubated at 30°C for 10 min. Cells were centrifuged and resuspended in 150 μl of −Met medium without ammonium sulfate, and radiolabeled with [^35^S]-Met (40 μCi per 1.5 OD_600_ units of cells) for 5 min at 30°C. After incubation, labeling was stopped by the addition of 750 μl of ice-cold stop solution buffer containing 20 mM Tris-HCl (pH 7.5) and 20 mM sodium azide. Cell pellets were harvested by centrifugation (16,000 ***g***, 1 min, 4°C) and stored at −20°C until use.

For pulse-chase experiments, 1.5 OD_600_ units of cells were harvested for each time point. Cells were prepared the same way as for pulse labeling, except that cells were resuspended in −Met medium of twice or three times the volume, corresponding to the number of time points for chase. Radiolabeling was stopped and chased by the addition of 50 μl of 200 mM nonradioactive Met medium per 1.5 OD_600_ units of cells for each time point. The reaction was stopped by transferring 1.5 OD_600_ units of cells to 750 μl of ice-cold stop solution buffer followed by centrifugation, and the cell pellets were kept frozen at −20°C until use.

### Tunicamycin treatment

Tunicamycin treatment of growing cells for radiolabeling and autoradiography was carried out as described previously (Yim et al., 2018). Briefly, prior to radiolabeling, cells were starved with 1 ml of −Met medium without ammonium sulfate for 30 min at 30°C in the presence of 100 μg/ml tunicamycin (Sigma) dissolved in DMSO, while control cells were mock-treated with DMSO.

### Immunoprecipitation and SDS–PAGE

Radiolabeled cell pellets were resuspended in 100 μl of lysis buffer [20 mM Tris-HCl (pH 7.5), 1% SDS, 1 mM DTT, 1 mM PMSF, and 1× Protease Inhibitor Cocktail (Quartett)] and mixed with 100 μl of ice-cold glass beads. Cell suspensions were vortexed for 2 min twice, keeping the samples on ice for 1 min in between. Subsequently, the samples were incubated at 60°C for 15 min and centrifuged (6000 ***g***, 1 min, 4°C). Supernatant fractions were mixed with 500 μl of immunoprecipitation buffer [15 mM Tris-HCl (pH 7.5), 0.1% SDS, 1% Triton X-100, and 150 mM NaCl], 1 μl of anti-HA antibody (MMS-101R; Biolegend) and 20 μl of prewashed protein G–agarose beads (Thermo Fisher Scientific, Pierce) and rotated at room temperature for 3 h. The agarose beads were washed twice with immunoprecipitation buffer, once with ConA buffer [500 mM NaCl, 20 mM Tris-HCl (pH 7.5) and 1% Triton X-100], and once with buffer C [50 mM NaCl and 10 mM Tris-HCl (pH 7.5)]. The beads were incubated with 50 μl of SDS sample buffer [50 mM DTT, 50 mM Tris-HCl (pH 7.6), 5% SDS, 5% glycerol, 50 mM EDTA, 1 mM PMSF, 1× Protease Inhibitor Cocktail (Quartett) and Bromophenol Blue] at 60°C for 15 min, followed by Endo H (Promega) treatment at 37°C for 1 h. Protein samples were then loaded onto SDS–PAGE gels and separated by electrophoresis.

### Data quantification

A Typhoon FLA 7000 phosphoimager and a Typhoon FLA 9500 phosphoimager were used for the detection of radiolabeled signals on SDS–PAGE gels by autoradiography. Data were processed and quantified using MultiGauge version 3.0 software (Fujifilm). Cleavage efficiency was calculated from the band intensities of glycosylated bands using the following formula: 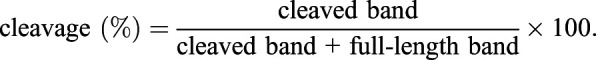


Cell growth assay and carbonate extraction samples were detected using a ChemiDoc XRS+, and resulting data were processed using Image Lab software (Bio-Rad).

### Statistical analysis

Statistical analyses of obtained quantification data were performed using Microsoft Excel 2013 or GraphPad Prism 8 for Windows.

### Carbonate extraction

Carbonate extraction was carried out as described previously (Yim et al., 2018), with the following modifications. Five OD_600_ units of cells were harvested by centrifugation (2170 ***g***, 5 min, 4°C), washed with distilled H_2_O and subjected to lysis. The final lysate was subjected to centrifugation (20,000 ***g***, 30 s, 4°C) to remove cell debris and transferred to a new pre-chilled tube. Centrifugation (20,000 ***g***, 20 min, 4°C) followed after incubation with 0.1 M Na_2_CO_3_ (pH 11.5). Trichloroacetic acid (TCA)-precipitated ‘total’, ‘supernatant’ and ‘pellet’ fractions were centrifuged (20,000 ***g***, 15 min, 4°C) and washed with acetone. Samples were resuspended in SDS sample buffer and analyzed by SDS–PAGE and western blotting. Mouse anti-HA primary antibody (1:10,000 dilution; MMS-101R, Biolegend) and horseradish peroxidase-conjugated anti-mouse secondary antibody (1:30,000 dilution; NCI1430KR, Thermo Fisher Scientific) were used for immunodetection.

### *In vitro* transcription and translation

For *in vitro* protein synthesis, the TnT Quick Coupled SP6 Transcription/Translation System (Promega) was used by following the manufacturer's protocol. The pGEM-4Z plasmid containing ΔSS-CPY (signal sequence of CPY deleted) was incubated with TnT SP6 Quick Master Mix and [^35^S]-Met for 1 h at 30°C. The synthesized proteins were then analyzed by SDS–PAGE and autoradiography.

### Mass spectrometry analysis of the abundance of SPC subunits in WT and *spc1*Δ strains

Cells from the W303-1α and *spc1*Δ strains were grown overnight in YPD medium (1% yeast extract, 2% peptone and 2% glucose) at 30°C in biological triplicates. Fifteen OD_600_ units of cells were harvested for each strain and subjected to cell lysis by vortexing for 10 min at 4°C with 200 µl of lysis buffer [8 M urea, 1× PIC (Protease Inhibitor Cocktail, Quartett) and 1 mM PMSF] and glass beads. The resulting cell lysates were reduced and alkylated with 10 mM DTT and 40 mM iodoacetamide, respectively, followed by trypsinization after 10-fold dilution with 50 mM ammonium bicarbonate buffer. The digested samples were then subjected to further clean-up with a C18 cartridge (Discovery DSC-18 SPE tube; SUPELCO, 52601-U).

For quantitative analysis, 10 μg of the biological triplicates of individual samples was subjected to TMT labeling (TMT10plex Isobaric Label Reagent Set, 1×0.8 mg; Thermo Fisher Scientific, 90111) as follows: TMT-126, 128N, and 130C for W303-1α samples; TMT-127C, 129N, and 130N for *spc1*Δ samples. The TMT-labeled peptide sample was subjected to LC-MS3 analysis using an Orbitrap Fusion Lumos (Thermo Fisher Scientific) with the following mass spectrometric parameters. The ten most intense ions were first isolated at 0.5 Th precursor isolation width under identical full MS scan settings for CID MS2 in an ion trap (ITmax 150 ms and AGC 4E3). The ten most intense MS2 fragment ions were synchronously isolated for HCD MS3 (AGC 1.5E5, ITmax 250 ms, and NCE 55%) at an isolation width of 2* m*/*z*. Data were analyzed using MaxQuant 1.6.2.3, based on Uniprot_swissprot protein database (*Saccharomyces cerevisiae*, 2020.12.02 release).

### Co-immunoprecipitation

Experimental procedures were based on those described previously (Zhang et al., 2017), with the following modifications. Crude membrane was isolated from ∼15 OD_600_ units of cells and solubilized with 400 μl of lysis buffer [50 mM HEPES-KOH in phosphate-buffered saline, pH 6.8, 1% Triton X-100, 150 mM KOAc, 2 mM Mg(OAc)_2_, 1 mM CaCl_2_, 15% glycerol, 1× PIC (Quartett), 2 mM PMSF] by rotation at 4°C for 1 h. After centrifugation at 20,238 ***g*** for 30 min at 4°C, the soluble fraction was transferred to a tube containing 25 μl of protein G–agarose beads prewashed three times with lysis buffer, followed by rotation for 30 min at 4°C. Beads were removed by quick centrifugation (10,000 ***g***), and 15 μl of the lysate was saved as the ‘Input’ fraction, while all the remaining supernatant was transferred to a new tube containing 25 μl of prewashed protein G–agarose beads and 1 μl of anti-FLAG mouse antibody (014-22383, FUJIFILM Wako Pure Chemical Corporation). The immunoprecipitation mixture was rotated for ∼4 h at 4°C. Beads were washed three times with lysis buffer and sampled by incubation with 40 μl of SDS sample buffer for 15 min at 55°C as the ‘IP’ fraction. The ‘Input’ fraction was mixed with 65 μl of SDS sample buffer and incubated for 15 min at 55°C.

## Supplementary Material

Supplementary information
