# Supplementary material for: Spc1 regulates the signal peptidase-mediated processing of membrane proteins
Source: J Cell Sci. 2021 Jul 9;134(13):jcs258936. doi: 10.1242/jcs.258936 (PMC8277137; doi:10.1242/jcs.258936)
Supplement: Supplementary information [file joces-134-258936-s1.pdf]

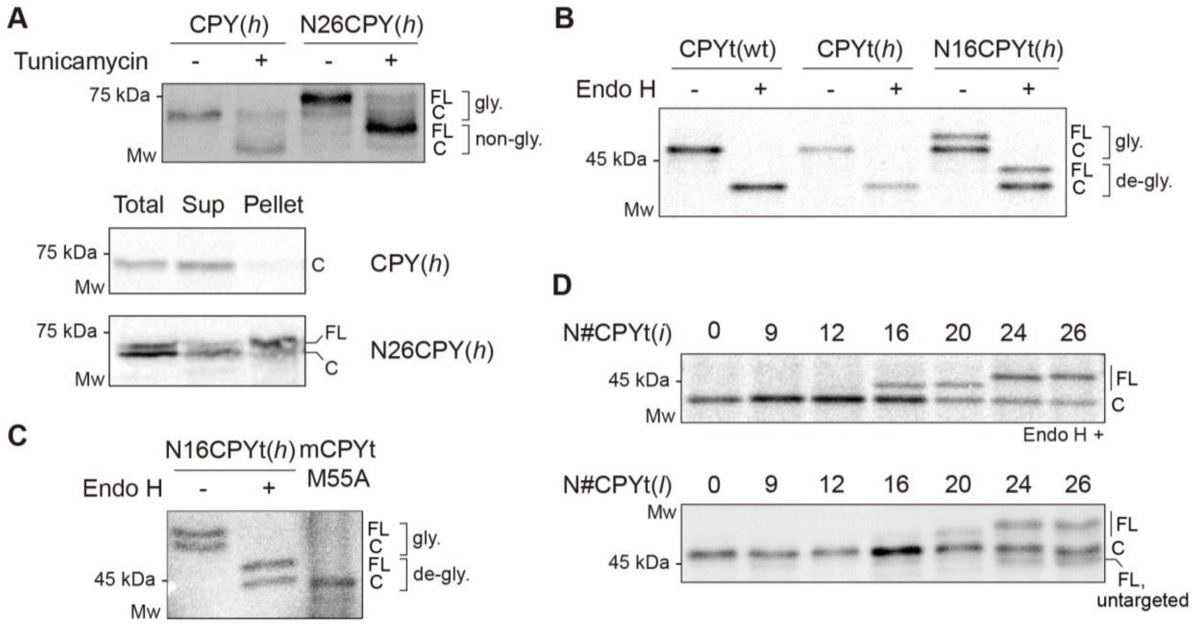

Figure S1. SPase-mediated processing of CPY variants depends on the N-length and hydrophobicity of the SS. (A) *Top*, CPY(*h*) and N26CPY(*h*) constructs in WT cells were radiolabeled for 5 min at 30°C in the presence or absence of tunicamycin, followed by immunoprecipitation and SDS-PAGE and analysis by autoradiography. *Bottom*, carbonate extraction was carried out. Sup, supernatant. (B) CPYt(wt), CPYt(*h*), and N16CPYt(*h*) constructs in WT cells were radiolabeled for 5 min at 30°C and subjected to immunoprecipitation for protein sampling. All protein samples were treated with Endo H prior to SDS-PAGE and analyzed by autoradiography. (C) N16CPYt(*h*) in the WT strain was radiolabeled for 5 min at 30°C, and the resulting protein sample was compared with the *in vitro* translated SS-deleted mature CPY (mCPYt M55A) on an SDS-PAGE gel. mCPYt M55A, in which M55 was substituted to alanine to silent the alternative start codon. (D) N#CPYt(*i*) (top) and N#CPYt(*l*) (bottom) variants in WT cells were analyzed as in Fig. 1B. In the N#CPYt(*l*) variants, a minor amount of the unglycosylated band was detected when the N-length became longer than 24, indicating inefficient translocation.

Journal of Cell Science • Supplementary information

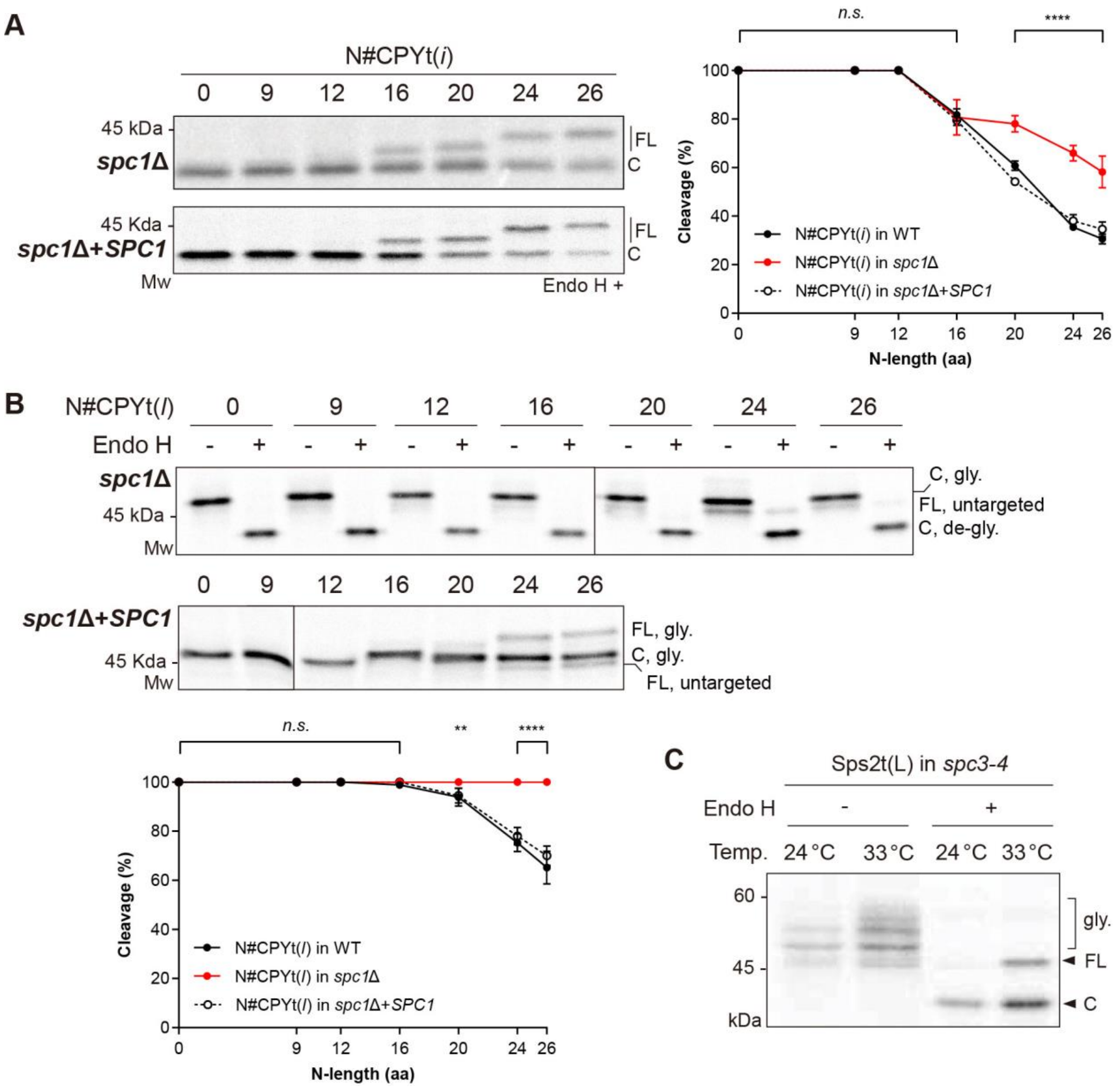

Figure S2. Cleavage of internal SSs is increased in the absence of Spc1. (A) N#CPYt(*i*) variants in *spc1*Δ and *spc1*Δ+*SPC1* cells were expressed, and protein samples were prepared as in Fig. 1B. Cleavage was analyzed as in Fig. 1F, and the data from the WT, *spc1*Δ and *spc1*Δ+*SPC1* strains were compared. (B) N#CPYt(*l*) variants in the WT, *spc1*Δ and *spc1*Δ+*SPC1* strains were analyzed, and the data were compared as in (A). p-values were calculated by multiple t-tests; *n.s.*, p>0.05; **, p≤0.01; ****, p≤0.0001. (C) Processing of Sps2t variants in the *spc3-4* strain at 24°C or 33°C.

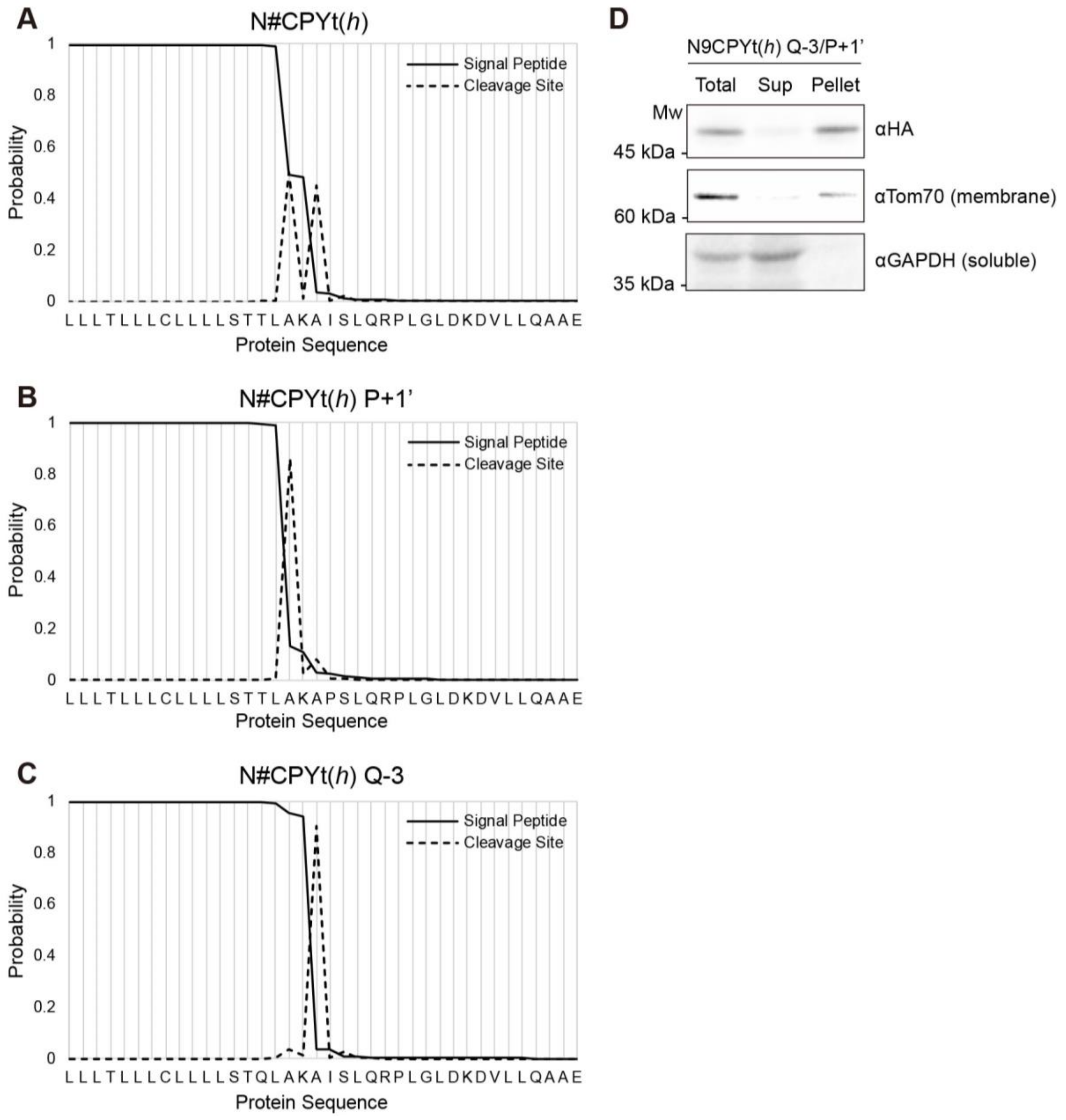

Figure S3. Prediction of cleavage sites in CPY SSs. Cleavage sites were predicted using SignalP 5.0 software (http://www.cbs.dtu.dk/services/SignalP/) (Almagro Armenteros et al., 2019). (A) N#CPY(*h*), (B) N#CPY(*h*) P+1', (C) N#CPY(*h*) Q-3. Peaks of the dashed line indicate the predicted cleavage sites. (D) Carbonate extraction of N9CPYt(*h*) Q-3/ P+1'. Sup, supernatant fraction. Anti-Tom70 and anti-GAPDH antibodies were used as controls for membrane and soluble proteins, respectively.

Journal of Cell Science • Supplementary information

Figure S4

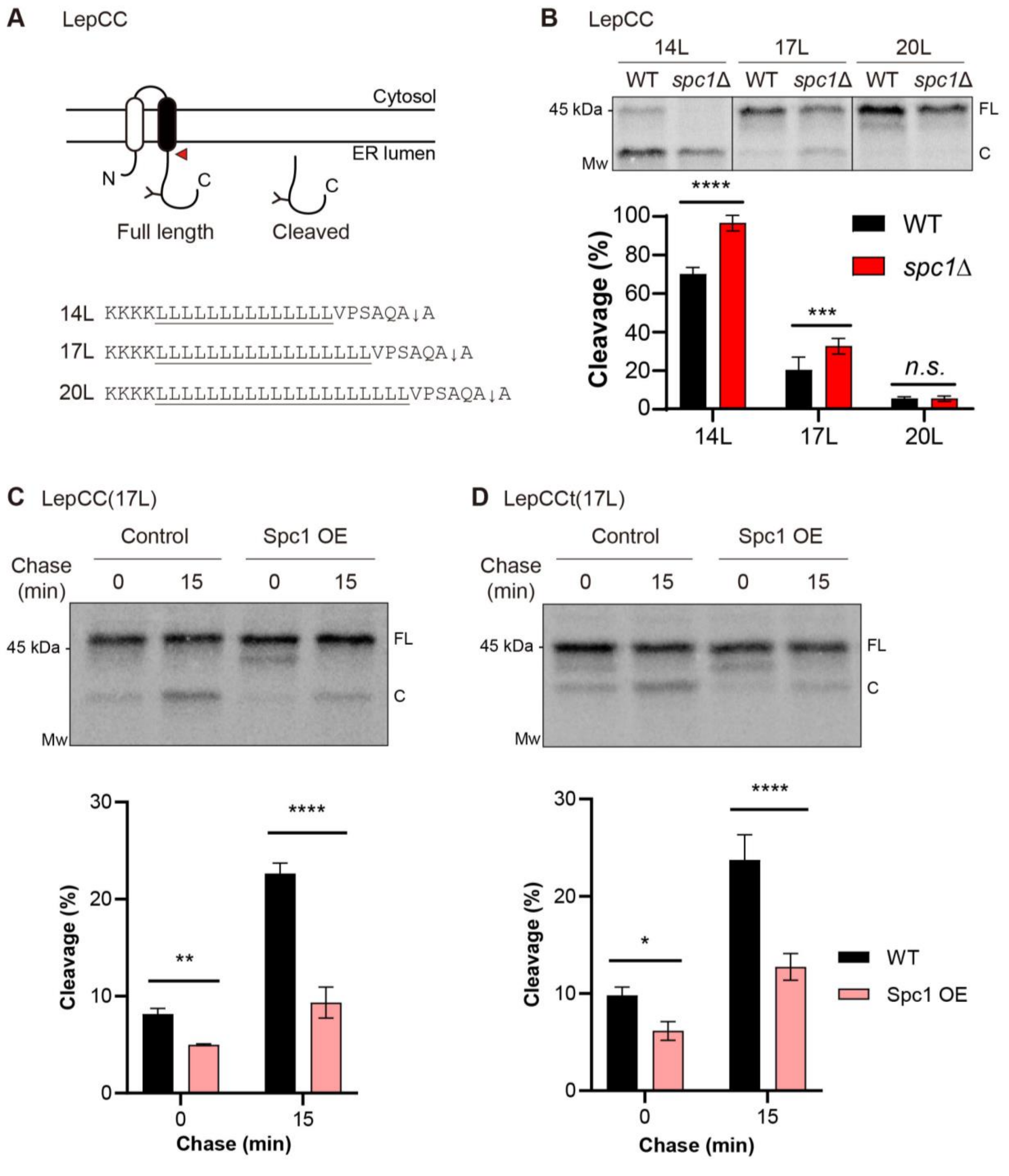

Journal of Cell Science • Supplementary information

Figure S4. SPase-mediated processing of membrane proteins is modulated by Spc1. (A) Schematics of LepCC. The H segment is colored black, and an N-linked glycosylation site is indicated as Y. Flanking and TM sequences of an H segment are shown for three LepCC variants. The cleavage site is shown as an arrow (↓). A red arrowhead points to cleavage by SPase. (B) LepCC variants in the WT or *spc1*Δ strain radiolabeled for 5 min at 30°C were analyzed as shown in Fig. 4. At least three independent experiments were carried out. The representative blot is shown (*top*) and the average is shown with the standard deviation (*bottom*). (C and D) LepCC(17L) (C) or LepCCt(17L) (D) in WT cells harboring control vector or Spc1 overexpression (OE) vector. Transformants were subjected to radiolabeling for 5 min at 30°C followed by chasing for the indicated time points. Cleavage (%) was calculated as in Fig. 1F. For all the experimental sets, three independent experiments were carried out, and the average is shown with the standard deviation. FL, full length; C, cleaved. p-values were calculated by multiple t-tests; *n.s.*, $p>0.05$; *, $p\leq0.05$; **, $p\leq0.01$; ***, $p\leq0.001$; ****, $p\leq0.0001$.

Journal of Cell Science • Supplementary information
